# Honeycomb-like ZnO Mesoporous Nanowall Arrays Modified with Ag Nanoparticles for Highly Efficient Photocatalytic Activity

**DOI:** 10.1038/s41598-017-11100-8

**Published:** 2017-09-14

**Authors:** Yimeng Feng, Guojing Wang, Jiecui Liao, Wei Li, Chienhua Chen, Mingyang Li, Zhengcao Li

**Affiliations:** 1 State Key Lab of New Ceramic and Fine Processing, School of Materials Science & Engineering, Tsinghua University, Beijing, 100084 China; 2 Key Lab of Advanced Materials (MOE), School of Materials Science and Engineering, Tsinghua University, Beijing, 100084 China; 3 Department of Engineering Physics, Tsinghua University, Beijing, 100084 China

## Abstract

A new structure of honeycomb-like ZnO mesoporous nanowall arrays (MNWAs) with highly efficient photocatalytic activity was designed and successfully synthesized on Al foil by hydrothermal method. The nanowalls of ZnO-MNWAs have mesopores, which possess a large surface area. The visible light absorption of ZnO-MNWAs was efficiently stronger than ZnO nanowire, resulting in that the photocatalytic activity of ZnO-MNWAs, whose bandgap energy was 3.12 eV, was 5.97 times than that of ZnO nanowires in the degradation of methyl orange. Besides, Al foil acted as a good electron conductor which was beneficial to the separation of photo-induced electron-hole pairs. After modifying ZnO-MNWAs with a proper amount of Ag nanoparticles (NPs), photocatalytic activity could be further enhanced. The photocatalytic activity of ZnO-MNWAs with the optimal amount of Ag NPs was 9.08 times than that of ZnO nanowires and 1.52 times than that of pure ZnO-MNWAs.

## Introduction

Recently, the industrial pollution has become a serious public problem, as a variety of dyes in polluted water result in poisoning to human and the environment^1^. Organic pollutants can be transformed into nontoxic resultants by low-cost, easily-obtained and high-performance methods. For example, photocatalysis^1–12^ and electrochemical degradation^13^ have drawn researchers’ attention. Especially, photocatalysis have been widely applied^14–16^.

The typical n-type semiconductor, ZnO, contributes to its widespread use in not only photocatalytic applications such as the degradation of organic pollution^2–4, 8, 11, 12^, the generation of H_2_^17, 18^ and the reduction of CO_2_^5^, but also gas sensors^15, 19, 20^, solar cells^21^ and memory devices^22^. ZnO has a wide and direct bandgap energy which is about 3.37 eV and has a large room temperature exciton binding energy (60 meV)^3, 8, 23^. It has been broadly applied in photocatalysis due to its photosensitivity, catalytic properties, high charge mobility rate and low cost^7, 8, 11, 17^. However, ZnO has some disadvantages when serving as a photocatalyst. Firstly, ZnO can only make use of the ultraviolet light (*λ* < 400 nm) which only consists 5% of the energy of sunlight^2–4, 17^. Moreover. ZnO has a high recombination ratio of photo-induced electron-hole pairs during photocatalytic process. Finally, ZnO faces with a severe light corrosion^17, 24^, making it inapplicable as a photocatalyst.

In order to solve the above-mentioned problems, firstly, ZnO can be synthesized with new nanostructures^4, 11^ to narrow energy bandgap or to offer large surface area. Nanostructures, including flower-like^4^, rod-shaped and belt-shaped^5^, sea urchin-shaped^11^ and hollow pencil-like^25^, can have a significant promotion on the efficiency of photocatalysis activity. However, photocatalysts with nanoparticle are impractical to reuse. Then new nanoarray structures of materials are synthesized on the different substrate for good reuse, such as silicon^3^, brass foils^2^, nickel foam^26, 27^ and so on. Highly ordered structures of materials are mostly synthesized by photolithography^28^ or with template-assisted^29, 30^, and porous films are always synthesized by an electrodeposition process^31^. Though these materials in recent works have excellent photocatalytic activities, including mesoporous TiO_2_ with CdS quantum dots^32^, self-floating TiO_2_ foams with 3D macro-mesoporous architectures^33^ and ZnO nanorod array with a mesoporous surface mediated by Cd^2+^ ^34^. These synthesis methods are too complicated and high cost, so a simple method without template is favoured. For instance, heterojunctions have been synthesized by simple hydrothermal method^3, 35^. Finally, to overcome the rapid recombination of electron-hole pairs, reports verify that ZnO modified with metals elements, such as Cu^1, 4^, Co^20^, Pt^36^, Au^37^ and Ag^7, 9, 38^, can significantly enchance photocatalytic activity by offering electron sinks. The electrons in the conduct band (CB) of the semiconductor can transfer to the electron sinks and the holes remain in the valence band (VB) of semiconductor. The Schottky barrier at the metal-semiconductor interface can contribute to the separation of electron-hole pairs^16, 39^. Ag is one of the most common metal to modify with semiconductors photocatalysts. Because not only Ag is much cheaper than Au and Pt, but also it has more significant enhancement on photocatalytic activity than Cu and Co^38^.

In this work, we have developed a new honeycomb-like ZnO mesoporous nanowall arrays (MNWAs) structure on Al foil substrate by simple hydrothermal method. The honeycomb-like ZnO-MNWAs were then modified with Ag NPs, and the optimal amounts of Ag NPs was investigated. The photocatalytic activity of the degradation of organic dyes by the synthesized materials was measured under visible light illumination, and the surface morphologies and chemical properties were characterized. Besides, the possible enhancement mechanism of photocatalytic activity was also discussed based on the utilization of visible light and the separation mechanism of the electron-hole pairs.

## Results and Discussion

### Morphology and structures

**Figure 1:**
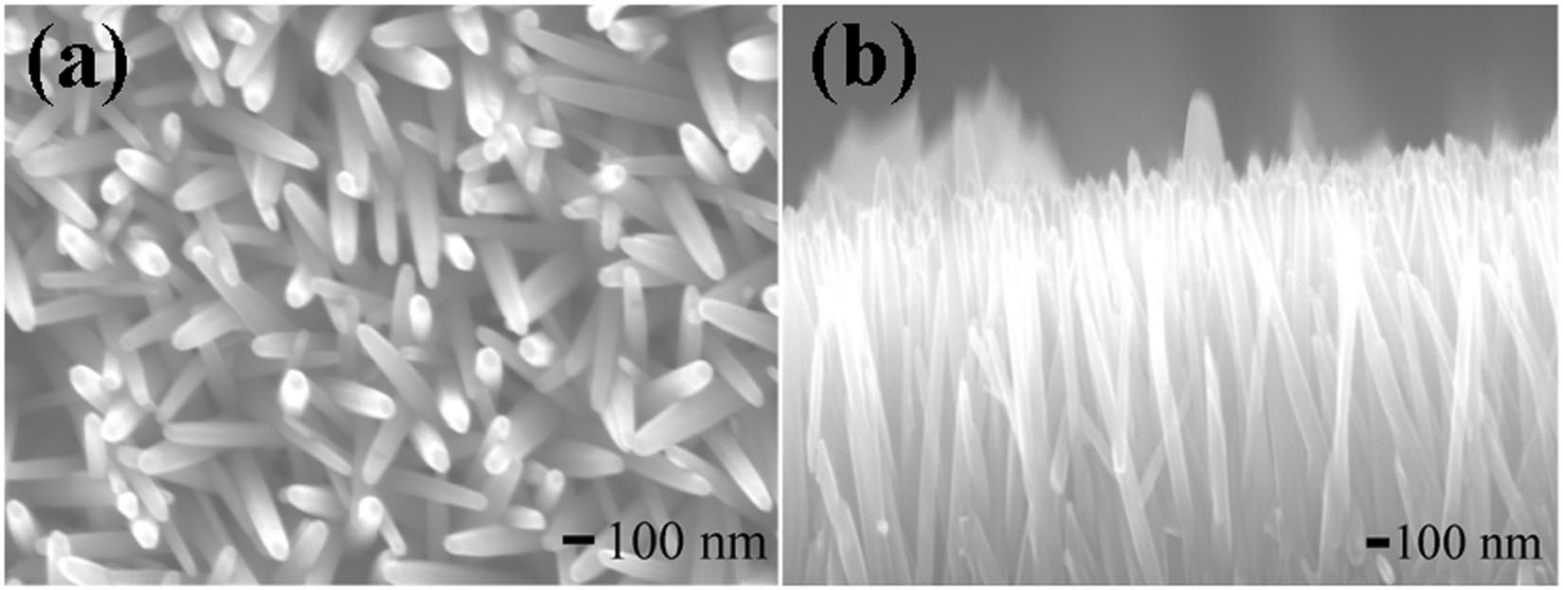
FE-SEM images of ZnO nanowires with (**a**) the top view; (**b**) the sectional view.

The morphology of ZnO nanowires were characterized by FE-SEM, and Fig. 1(a) and (b) are the top view and sectional view of ZnO nanowires. These images clearly show that ZnO nanowires had good discrete structures. The diameter of ZnO nanowires was less than 100 nm and the length of ZnO nanowire was about several micrometers. From Fig. 1(a) and (b), it can be clearly seen that these nanowires all are uniform.

**Figure 2:**
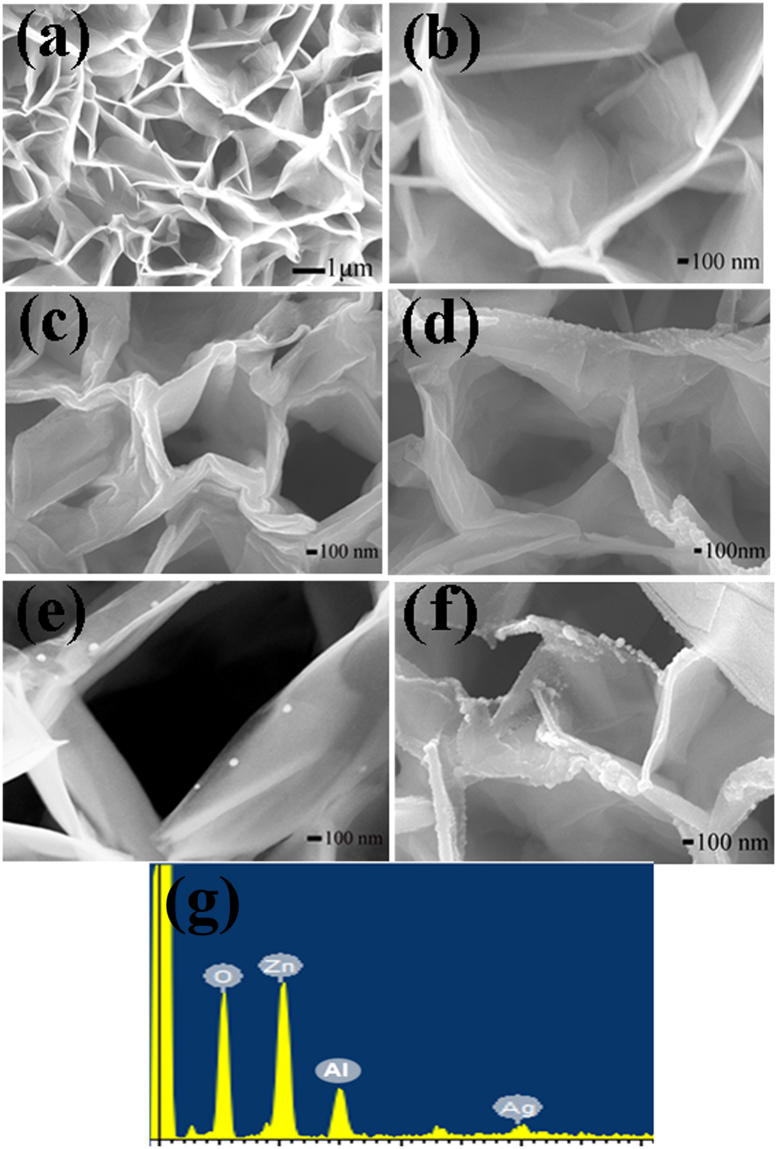
FE-SEM images of (**a**)large area of ZnO-MNWAs; (**b**) partial area of ZnO-MNWAs, (**c**) 40s-Ag@ZnO-MNWAs, (**d**) 45s-Ag@ZnO-MNWAs, (**e**) 50s-Ag@ZnO-MNWAs, (**f**) 60s-Ag@ZnO-MNWAs; (**g**) EDS images of 45s-Ag@ZnO-MNWAs.

Figure 2(a) and (b) are FE-SEM images of large area and partial area ZnO-MNWA, respectively. The diameter of holes of honeycomb was in the range of 1~2 μm. The morphology of ZnO-MNWAs on the Al foil was different with that of ZnO nanowires on the Si substrate, because Al element in the substrate could turn ZnO into nanoplane rather than nanowire. As the hydrothermal process went on, the ZnO nanoplanes would self-assembled into honeycomb-like microstructure^15^. According to Fig. 2(a), ZnO-MNWAs had a well-defined 3D microstructure, and it was composed by nanowalls whose thickness was about 100 nm. Figure 2(c)~(f) depict the microscopic morphology and crystalline regularity of ZnO-MNWAs modified with different amounts of Ag NPs. Ag NPs amounts increased along with the sputtering time, but their sizes all were less than 100 nm. 40 s-Ag@ZnO-MNWAs almost showed no Ag NPs on the surface, while Ag NPs were deposited uniformly on the surface of 45 s-Ag@ZnO-MNWAs. When sputtering times were longer than 45 s, a significant phenomenon of Ag NPs agglomeration occurred in 50 s-Ag@ZnO-MNWAs and 60 s-Ag@ZnO-MNWAs. Figure 2(g) shows the EDS pattern of 45 s-Ag@ZnO-MNWAs and the elements were exhibited in Table 1. Besides the Al element from Al foil, there were elements of Zn, O and Ag. Compared with the amount of Zn and O, the amount of Ag was only 0.76%. Therefore, 45 s-Ag@ZnO-MNWAs is primarily composed by Zn, O elements and a slight amount of Ag elements. The element mappings of 45 s-Ag@ZnO-MNWAs are shown as Figure S1 in Supplementary information. It shows that the elements of Zn, O and Ag all have a uniform distribution.

**Table 1:** The amount of elements in 45 s-Ag@ZnO-MNWAs.

Elements	Weight Ratio (%)	Atomic Percentage (%)
O	46.63	78.50
Zn	50.32	20.74
Ag	3.05	0.76
Total	100.00	100.00

**Figure 3:**
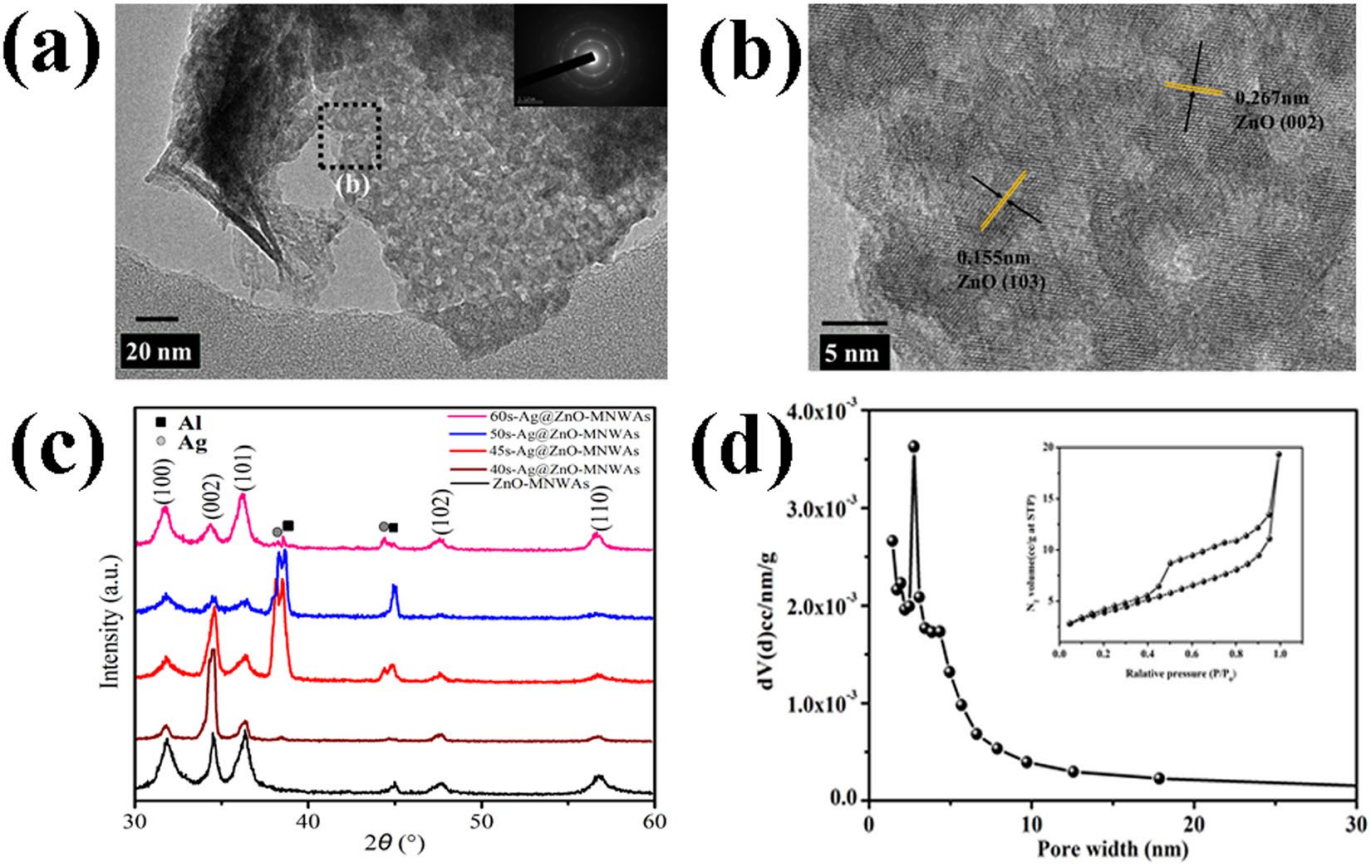
FE-TEM images of 45s-Ag@ZnO-MNWAs in (**a**) large area, (**b**) partial area ;(**c**) XRD patterns of all samples; (**d**) N2 absorption and desorption isotherms and the pore size distribution of 45s-Ag@ZnO-MNWAs.

Figure 3(a) and (b) is FE-TEM images of 45 s-Ag@ZnO-MNWAs. Figure 3(a) and (b) show that 45 s-Ag@ZnO-MNWAs were actually composed by nanoplanes which contained lots of mesopores. Abundant mesopores enhance light absoption ability and the accessibility of dye absorption^40^. Furthermore, the diameter of the mesopores was around 2~6 nm. Significantly, mesopores in nanowalls further expand surface area for degradation reaction. Distances between two adjacent planes were 0.267 nm and 0.115 nm corresponding to plane (002) and (103) of ZnO, suggesting a polycrystalline crystal structure of ZnO-MNWAs. The interplanar spacing of 45 s-Ag@ZnO-MNWAs is slightly larger than ZnO bulk. According to the first-principles calculations, substitutional-Ag to surface-Zn atom is the most energetically favorable^41^. Therefore, the ionic radius of Ag^+^ (1.15 Å) is larger than that of Zn^2+^ (0.74 Å) which makes lattice distortion^32, 42^. The ring electron diffraction pattern in inserts of Fig. 3(a) also indicates a polycrystalline hexagonal crystal structure of ZnO-MNWAs^8^. The interplanar spacing of Ag NPs was not observed in TEM patterns, because the amount of Ag NPs was too little.

Figure 3(c) shows the XRD patterns of all samples in the range of 30~60°. The peaks of ZnO-MNWAs were located at 31.81°, 34.50°, 36.37°, 47.71°, 56.77°, corresponding to the (100), (002), (101), (102), (110) planes of hexagonal ZnO structure (JCPDS files 89–1397)^5^, respectively. After modifying Ag NPs, all peaks slightly shifted to lower angles, meaning Ag substituted Zn atom^43^. The peak intensity and width of ZnO-MNWAs in XRD pattern significantly prove that it was a polycrystalline nanostructure^42^, which in accord with The ring electron diffraction pattern in Fig. 3(a). There were no characteristic peaks of Ag in 40 s-Ag@ZnO-MNWAs to be observed in XRD pattern. When modified ZnO-MNWA with Ag NPs for 45 s, 50 s and 60 s, the characteristic peaks of Ag located at 38.1° and 44.3° were observed, which were corresponding to planes of (111) and (200), respectively. Therefore, Ag NPs is formed in ZnO-MNWA when the sputterring times were proper. Figure 3(d) is the N_2_ absorption and desorption isotherms and the pore size distribution of 45 s-Ag@ZnO-MNWAs. From Fig. 3(d), it shows that a type IV-like isotherm has a sharp increase of the absorbed volume at P/P_0_ = 0.65, which means the nanowalls of 45 s-Ag@ZnO-MNWAs have well-developed mesopores. The pore size distribution was calculated based on Barrett-Joyner-Halenda theory^44^, and the narrow pore-size distribution with an average pore size of 2.76 nm was presented in an inset picture of Fig. 3(d). The average pore size was in accord with the TEM picture in Fig. 3(b). The BET surface area and specific pore volume of 45 s-Ag@ZnO-MNWAs were 41.775 m^2^/g and 299.9 cc/g.

### XPS analysis

**Figure 4:**
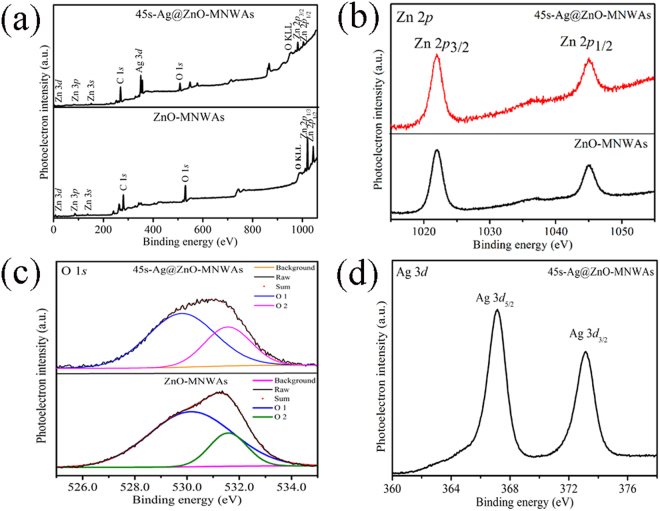
XPS spectra of ZnO-MNWAs and 45s-Ag@ZnO-MNWAs.

**Figure 5:**
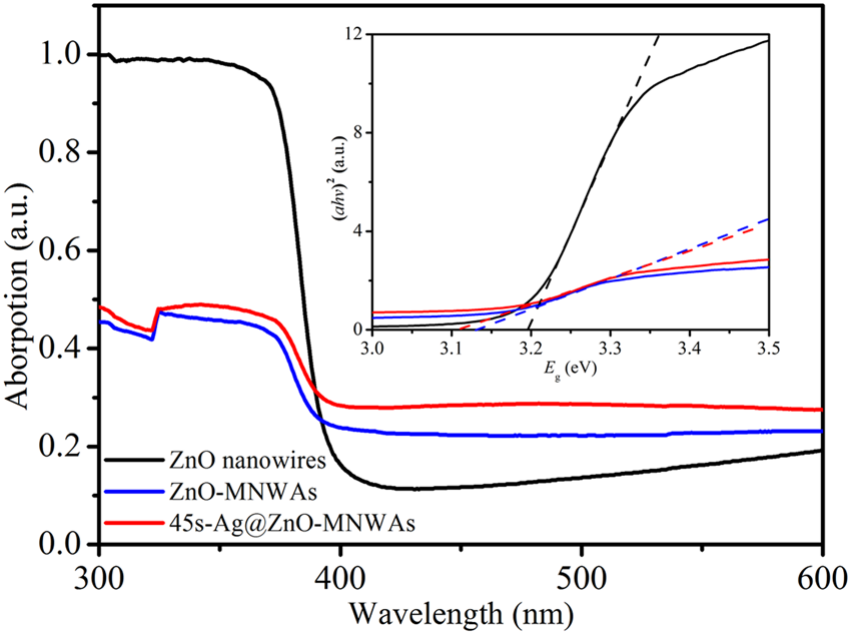
UV-Vis spectra of ZnO NRs, ZnO-MNWAs and 45s-Ag@ZnO-MNWAs.

XPS analysis was used to analyze chemical states and surface composition of ZnO-MNWAs and 45 s-Ag@ZnO-MNWAs, as shown in Fig. 4. The energy scale was calibrated with the C 1 *s* peak at 284.6 eV^21^. Figure 4(a) presents peaks of Zn and O elements are contained in ZnO-MNWAs, while peaks of Zn, O and Ag can be observed in the pattern of 45 s-Ag@ZnO-MNWAs. The Zn 2*p*_3/2_ and Zn 2*p*_1/2_ peaks of ZnO-MNWAs and 45 s-Ag@ZnO-MNWAs at 1022.0 eV and 1044.9 eV are presented in Figure 4(b) ^3^. According to Fig. 4(c), two separated O peaks whose banding energies (BE) are 530.2 eV and 531.6 eV were observed in both of ZnO-MNWAs and 45 s-Ag@ZnO-MNWAs. The component at 531.6 eV can be attributed to the OH groups on the surface of ZnO^8^, which obviously belongs to the absorbed H_2_O. And the BE of O at 530.0 eV belongs to Zn-O-Zn^7, 8^. Figure 5(d) shows Ag 3*d*_5/2_ and Ag 3*d*_3/2_ at 367.2 eV and 373.2 eV, which are slightly lower than the reported BE of Ag^+^ in substitutional lattice sites^10^. Electrons can easily transfer from Ag to the CB of ZnO, which result in that the peak of Ag moves to lower BE. It originates from that the work function of ZnO (5.20~5.30 eV) is larger than that of Ag (4.26 eV). Electron would transfer from Ag to the CB of ZnO until two systems get a Fermi energy level equilibrium, so a new fermi energy level was formed in the heterojunction of 45 s-Ag@ZnO-MNWAs^16, 38^.

### UV-Vis spectra

To investigate the enhanced effect of bandgap and light absorption on the photocatalytic activity of ZnO-MNWAs and 45 s-Ag@ZnO-MNWAs, UV-Vis spectra of samples is essential, as shown in Fig. 5. It depicts that the absorption edges of ZnO nanowires, ZnO-MNWAs and 45 s-Ag@ZnO-MNWAs all locate at around 400 nm, so a red shift is observed compared with ZnO bulk whose absorption edge is 380 nm. Because there exists the quantum confinement in nanomaterials^8^. Meanwhile, the visible light absorption of ZnO-MNWAs and 45 s-Ag@ZnO-MNWAs is significantly stronger than that of ZnO nanowires in the range of 400~600 nm. To calculate the bandgap energy, Equation (1) as follow were used^14^:

1 $$\alpha \,=K\cdot {(h{\rm{\nu }}-{E}_{g})}^{1/2}/h{\rm{\nu }}$$

where *α* is the absorption coefficient, *K* is a constant, *hν* is the photon energy, and *E*_g_ is the bandgap energy.

Therefore, the *E*_g_ of the samples could be acquired from the plot of (*αhν*)^2^ versus *hν*, shown as the insets in Fig. 5. Bandgap energy of ZnO nanowires was calculated as 3.20 eV, which was in accord with ZnO nanowires in previous research^2–4^. The bandgap energy of ZnO-MNWAs and 45 s-Ag@ZnO-MNWAs were calculated as 3.12 eV and 3.11 eV, respectively. The decrease of bandgap energy means that both of the new honeycomb nanostructure and modifying Ag NPs are helpful for the adjustment of bandgap. Therefore, ZnO-MNWAs and 45 s-Ag@ZnO-MNWAs offer more opportunities for the separation of photo-induced electron-hole pairs and enhance the absorption of visible light.

### Photoluminescence

**Figure 6:**
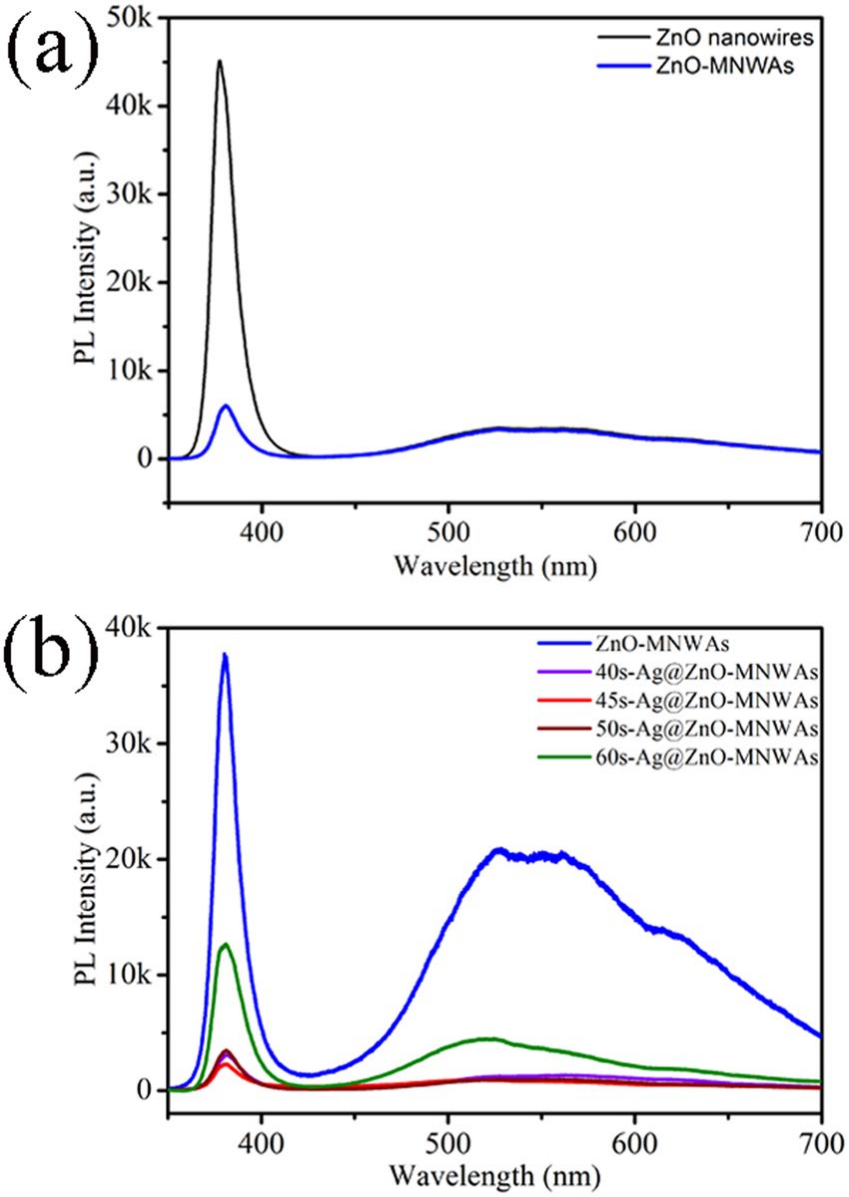
PL spectra of (**a**) ZnO nanowires and ZnO-MNWAs, (**b**) ZnO-MNWAs modified with different amounts of Ag NPs.

Photoluminescence (PL) study is essential to investigate electronic structure and photoelectric activity, as shown in Fig. 6. In Fig. 6(a), ZnO nanowires and ZnO-MNWAs both have strong ultraviolet emission peaks at 377 nm, which can be attributed to the recombination of induced electron-hole pairs. Obviously, PL intensity of ZnO-MNWAs significantly decreased compared with ZnO nanowires, as the recombination of free excitons were restricted. The broad emission peaks from 500 nm to 700 nm can be attributed to oxygen vacancies^3, 14^. Moreover, the PL spectra of ZnO-MNWAs with different amounts of Ag NPs are presented in Fig. 6(b). It shows that the PL intensity of ZnO-MNWAs with different amounts of Ag NPs was weaker than ZnO-MNWAs, because metal bandgap of Ag NPs acted as electron sinks and suppressed the recombination of electron-hole pairs. 45 s-Ag@ZnO-MNWAs has the most proper amount of Ag NPs, so its PL intensity is the weakest, revealing that it has the least recombination of electron-hole pairs. To further prove the photoelectrochemical c property of samples, photocurrent density-voltage (*J* –*V*) characteristics curves as Figure S2 in Supplementary information were measured under dark condition. *J-V* curves show that *V*_oc_ of ZnO nanowires and ZnO-MNWAs and 45 s-Ag@ZnO-MNWAs are 0.0631 V, 0.0724 V, 0.0827 V, respectively. These results show that photoelectrochemical activity was enhanced due to more effective separation of photogenerated electrons and holes in ZnO-MNWAs and 45 s-Ag@ZnO-MNWAs^45^.

### Photocatalytic activity

The first-order kinetics equation is simply given as following Equation (2):

2 $${{C}}_{{t}}={{\rm{C}}}_{{0}}\cdot \exp ({-}{k}\cdot {t})$$

where *t* is the time and *k* is the first-order constant, *C*_t_ is MO concentration when time is *t* and *C*_0_ is the original concentration.

**Figure 7:**
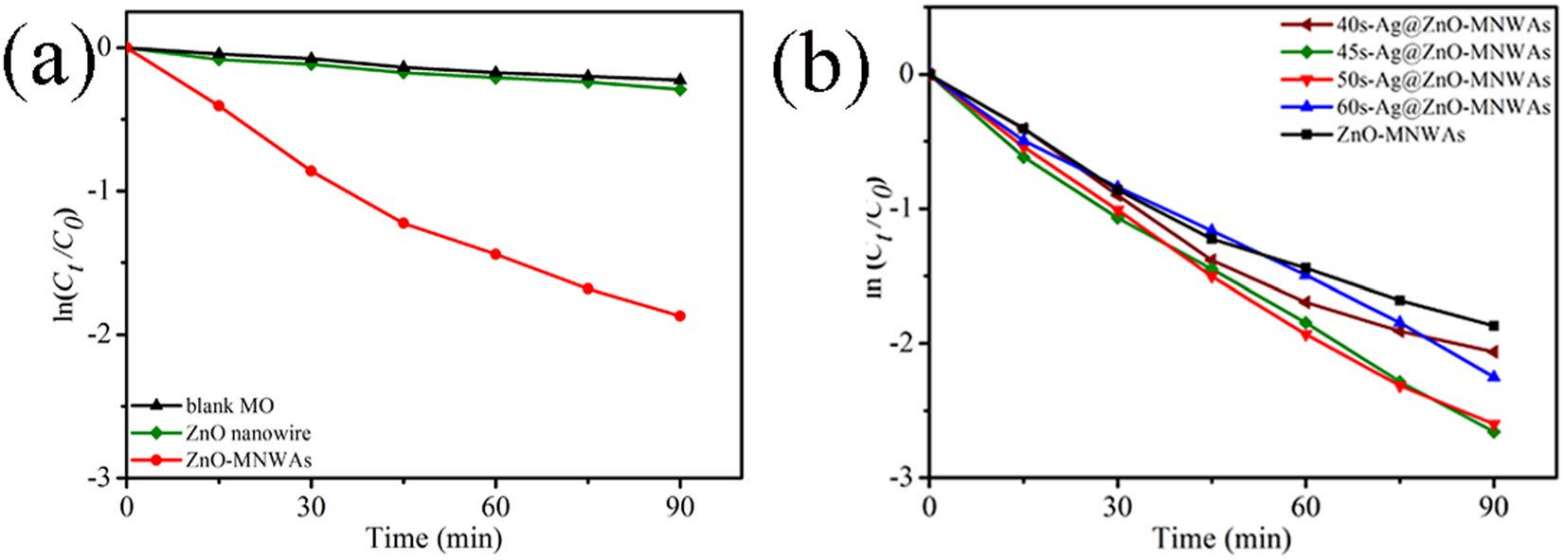
The photocatalytic activity of (**a**) ZnO nanowires and ZnO-MNWAs, (**b**) ZnO-MNWAs modifying with different amounts of Ag NPs.

According to Equation (2), the photocatalytic activity was plotted in Fig. 7. Figure 7(a) is the photocatalytic activity of ZnO-MNWAs and ZnO nanowires, and Fig. 7(b) is the photocatalytic activity of ZnO-MNWAs with different amounts of Ag NPs. The relationship between the degradation of MO and the illumination times is nearly linear. As the initial solution concentration of MO was lower enough and all sample was immersed in MO for 1 h in the dark to reach absorption equilibrium, so the degradation of ZnO nanowires, ZnO-MNWAs and ZnO-MNWAs with different amounts of Ag NPs all obey the first-order reaction kinetics according to Fig. 7 ^3, 14, 46–49^. The degradation percentages of ZnO nanowires, ZnO-MNWAs and Ag@ZnO-MNWAs with different amounts of Ag NPs were calculated from Figure S3 in Supplementary information. The first-order rate constants and the degradation percentages of samples are shown in Table 2. In term of the calculated first-order rate constants, the photocatalytic activity of ZnO-MNWAs and 45 s-Ag@ZnO-MNWAs are 5.97 times and 9.08 times than ZnO nanowires, respectively. From Fig. 7(b), the photocatalytic activity of 45 s-Ag@ZnO-MNWAs is 1.52 times than that of pure ZnO-MNWAs. The photocatalytic activities of samples degrading methylthionine chloride (MC) were presented as Figure S4 in Supplementary information. It shows that the efficiency of ZnO-MNWAs and 45 s-Ag@ZnO-MNWAs were 87.5% and 93.4% for degrading MC, respectively. Cycling tests of ZnO-MNWAs and 45 s-Ag@ZnO-MNWAs were performed under the visible light illumination, as Figure S5 in Supplementary information. ZnO-MNWAs and 45 s-Ag@ZnO-MNWAs remain 90% and 87% of their original photocatalytic activity, respectively.

**Table 2:** The first-order reaction rate constant *k* for all the examples.

Photocatalysts	*k* (min^−1^)	D (%)
ZnO nanowires	0.00325	25%
ZnO-MNWAs	0.01941	79%
40 s-Ag@ZnO-MNWAs	0.02157	87%
45 s-Ag@ZnO-MNWAs	0.02952	93%
50 s-Ag@ZnO-MNWAs	0.02879	92%
60 s-Ag@ZnO-MNWAs	0.02477	89%

### The photocatalytic mechanism

**Figure 8:**
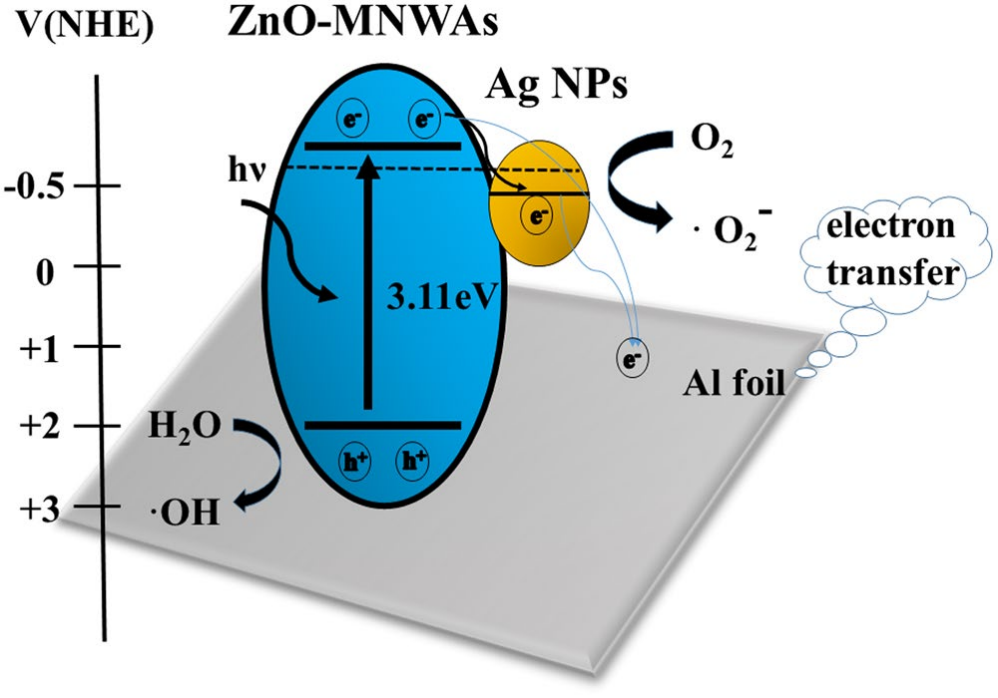
Simplified schematic diagram of the energy band structure and electron–hole pair separation in 45s-Ag@ZnO-MNWAs.

In this work, both of ZnO-MNWAs and 45 s-Ag@ZnO-MNWAs have significant enhancements in photocatalytic activity compared with ZnO nanowires. As for 45 s-Ag@ZnO-MNWAs nanocomposites on Al foil substrate, the CB and VB, presented as *E*_CB_ and *E*_VB_, are calculated by the following Equations (3) and (4) ^47^:

3 $${E}_{{\rm{VB}}}=X-{E}^{{\rm{e}}}+{\rm{0.5}}{E}_{g}$$

4 $${E}_{\mathrm{CB}}={E}_{{\rm{VB}}}-{E}_{g}$$

where X is the absolute electronegativity of the semiconductor, *E* ^e^ is the energy of free electrons on the hydrogen scale (*ca*. 4.5 eV), and *E*_g_ is bandgap energy. *E*_CB_ and *E*_VB_ were calculated as −0.38 eV and 2.73 eV, respectively. Figure 8 shows the possible photocatalytic mechanism of 45 s-Ag@ZnO-MNWAs. When 45 s-Ag@ZnO-MNWAs was illuminated by light whose energy is greater than its bandgap energy, photo-induced electrons (e^−^) would be excited from the VB to the CB, with the same amount of induced holes in VB. As the CB of ZnO-MNWAs is higher than the new Fermi energy level, induced-electron can transfer quickly from the CB of ZnO-MNWAs to Ag NPs. Therefore, modifying ZnO-MNWAs with a proper amount of Ag NPs can significantly enhance its photocatalytic activity, while too many Ag NPs in ZnO-MNWAs would suppress the photocatalytic activity. Ag bandgap acts as the electron-hole separation center when the amount is below the premium amount of Ag NPs. On the contrary, Ag NPs becomes new recombination centers when Ag amount is beyond the premium^38^. Al foil substrate is a good electron conductor, making it convenient for electrons to separate from holes in ZnO-MNWAs. Both of Al and Ag NPs contribute to the separation of induced electron-hole pairs, which is beneficial to maintain the amount of induced electron-hole pairs. Electrons can be trapped by the absorbed O_2_ on the surface of the sample, producing ·O_2_^−^ radical. While holes in VB react with H_2_O and produce hydroxyl radicals (·OH), which has a strong oxidation ability to degrade organic dye. These processes could be described as the following Equations (5)~(10) ^14^:

5 $${\rm{ZnO}}+h\nu \to {\rm{ZnO}}({{\rm{h}}}^{+})+{\rm{ZnO}}({{\rm{e}}}^{-})$$

6 $${\rm{ZnO}}({{\rm{h}}}^{+})+{{\rm{H}}}_{2}{\rm{O}}\to {\rm{ZnO}}+{{\rm{H}}}^{+}+\cdot {\rm{OH}}$$

7 $${\rm{ZnO}}({{\rm{e}}}^{-})+{{\rm{O}}}_{{\rm{2}}}\to {\rm{ZnO}}+\cdot {{{\rm{O}}}_{2}}^{-}$$

8 $$\cdot {{{\rm{O}}}_{2}}^{-}+{\rm{ZnO}}({{\rm{e}}}^{-})+{{\rm{H}}}^{+}\to {\rm{ZnO}}+{{\rm{H}}}_{2}{{\rm{O}}}_{2}$$

9 $${{\rm{H}}}_{2}{{\rm{O}}}_{2}+{\rm{ZnO}}({{\rm{e}}}^{-})\to \cdot {\rm{OH}}+{{\rm{OH}}}^{-}$$

10 $${\rm{Dye}}+\cdot {\rm{OH}}\to {\rm{Degradation}}\,{\rm{products}}$$

During the degradation process, organic dye pollutant can be successively attacked by active group ·OH and degrades to smaller molecules. Therefore, 45 s-Ag@ZnO-MNWAs have proper electron sinks of Ag NPs and is considered as the best visible light driven photocatalyst.

## Conclusion

In summary, a new honeycomb-like ZnO-MNWAs on Al foil was synthesized by low-cost hydrothermal method without template. Honeycomb-like ZnO-MNWAs were formed by mesoporous nanowall which offered large surface area. The experiments verified that the photocatalytic activity of new nanostructure ZnO-MNWAs was significantly enhanced to 5.97 times compared with ZnO nanowires, as ZnO-MNWAs have a narrower bandgap energy of 3.12 eV, which enhanced the absorption of visible light. Besides, ZnO-MNWAs offer enough surface areas for photocatalysis. In addition, Al substrate can transfer electron from ZnO-MNWAs which is helpful for the separation of photo-induced electron-hole pairs. ZnO-MNWAs modified with Ag NPs have further enhanced photocatalytic activity. After investigating the photocatalytic activity of ZnO-MNWAs with different mass contents of Ag NPs, the photocatalytic activity of 45 s-Ag@ZnO-MNWAs was found to be the best and was 9.08 times compared with that of ZnO nanowires. Besides, the photocatalytic activity of 45 s-Ag@ZnO-MNWAs is 1.52 times than that of pure ZnO-MNWAs. The good and stable photocatalytic activity was attributed to the adjustment of bandgap energy of 3.11 eV. Moreover, a proper amount of Ag NPs provides separation centers for electron-hole pairs and further enhances the photocatalytic activity. Therefore, honeycomb-like 45 s-Ag@ZnO-MNWAs is a potential visible-light-driven photocatalyst for the degradation of MO in pollutant water.

## Experimental

### Materials preparation

All chemicals used are reagent grade without further purification. Chemicals include zinc nitrate hexahydrate (Zn(NO_3_)_2_·6H_2_O), hexamethylenetetramine (HMT, C_6_H_12_N_4_), methylene orange (MO), methylthionine chloride (MC), acetone (CH_2_O), ethanol (C_2_H_6_O). The above materials are all commercially available.

### Preparation of ZnO-MNWAs and ZnO nanowires

Two kinds of ZnO with different morphologies were synthesized, including ZnO nanowires and ZnO-MNWAs. The former was grown on the silicon substrate while the later one was grown on the aluminum foil. The substrates were firstly cleaned in ultrasonic bath with acetone, ethanol and deionized water in turn. Then ZnO seed layer was deposited by radio frequency (RF) magnetron sputtering. The RF condition was as followings: the working vacuum was 0.6 Pa, gas flow was Ar 30 sccm, O_2_ was 10 sccm, RF electric power was about 70 W. Each sample was coated for 7 minutes. Afterwards, ZnO nanowires and ZnO-MNWAs were synthesized through a simple hydrothermal method on silicon substrate and aluminum foil at 95 °C for 4 h. Finally, all samples were annealed at 400 °C for 2 h.

### Preparation of Ag NPs modifying with ZnO-MNWAs

ZnO-MNWAs was decorated with Ag NPs by the direct-current (DC) magnetron sputtering. The DC conditions were as follows: the working vacuum was 1.0 Pa, Ar gas flow was 40 sccm, DC electric power was about 36 W. Each sample was respectively coated for 40 s, 45 s, 50 s and 60 s to investigate the effects of photocatalytic activity with different amounts of Ag NPs in ZnO-MNWAs. The samples are denoted as 40 s-Ag@ZnO-MNWAs, 45 s-Ag@ZnO-MNWAs, 50 s-Ag@ZnO-MNWAs and 60 s-Ag@ZnO-MNWAs. Then all these samples were annealed at 400 °C for 2 h.

### Characterizations

The morphologies, elements, and structures of samples in the experiment were investigated by filed-emission scanning electron microscopy (FE-SEM, JEOL-JSM 7001 F), energy disperse X-ray spectroscopy (EDS) and filed-emission transmission electron microscopy (FE-TEM, JEM-2010). The structures were characterized by X-ray diffraction (XRD: Rigaku Smart Lab) fixed in 0.5° with scanning rates of 8° min^−1^ in the 2*θ* range of 30~60°. The elemental and chemical states of the samples were evaluated by X-ray photoelectron spectroscopy (XPS, ESCALAB250Xi, Thermofisher Scientific). The photoluminescence spectra were recorded at room temperature by a Lab Ram HR Evolution Micro Raman Spectrometer. Nitrogen adsorption-desorption isotherms at 77 K were collected on an Quadrasorb SI-MP. The Brunauer–Emmett–Teller (BET) equation was used tocalculate the specific surface area. Pore size distributions were obtained using the Barrett–Joyner–Halenda (BJH) method from theadsorption branch of the isotherms. Photoelectrochemical properties were investigated by measuring the photocurrent intensity versus potential (*J-V* curve) using an electrochemistry workstation (CHI 660d, Chenhua Instrument). These measurements were carried out in a 250 mL quartz cell using a standard three-electrode configuration, composed of the samples as a working electrode, a Pt foil as a counter electrode, a saturated Ag/AgCl as a reference electrode, and 1 M KCl aqueous solution as the electrolyte.

### Photocatalytic activity

The photocatalytic tests of MO and MC were performed in a photoreaction chamber. The sample parameters were recorded by UV-Visible spectrophotometer (UV-2600, SHIMADZU), and the spectra was measured in the range of 300~600 nm.

The degradation of organic dyes was used to evaluate photocatalytic activity of samples. The size of ZnO samples was 1.5 × 1.5 cm^2^ and the concentration of MO solution used in the experiment was 10 μmol/L without pH buffer solution, while MC solution was 0.5 mg/L with PH buffer solution to PH 7. Each sample in organic dyes solution was firstly put in dark condition for 1 h to reach absorption equilibrium and then illuminated by a 50 W Xe light for 90 min. The light was filtered by a filter, so only visible light was applied in the degradation process. The photocatalytic activity was calculated according to the Lambert-Beer law^46^. The degradation rate was calculated by Equation (11)

11 $$D=\frac{{C}_{0}-{C}_{t}}{{C}_{0}}\approx \frac{{A}_{0}-{A}_{t}}{{A}_{0}}$$

where *C*_0_ and *A*_0_ are the MO concentration and absorption before illumination, and the concentration and absorption after illuminated are *C*_*t*_ and *A*_*t*_, respectively.

## Electronic supplementary material

Supplementary information
