# Supplementary material for: Honeycomb-like ZnO Mesoporous Nanowall Arrays Modified with Ag Nanoparticles for Highly Efficient Photocatalytic Activity
Source: Sci Rep. 2017 Sep 14;7:11622. doi: 10.1038/s41598-017-11100-8 (PMC5599540; doi:10.1038/s41598-017-11100-8)
Supplement: Supplementary file 1 — Supplementary information [file 41598_2017_11100_MOESM1_ESM.pdf]

Honeycomb-like ZnO Mesoporous Nanowall Arrays Modified with Ag Nanoparticles for Highly Efficient Photocatalytic Activity

Yimeng Feng[1], Guojing Wang[1], Jiecui Liao[1], Wei Li[1], Chienhua Chen[1], Mingyang Li[1,3], Zhengcao Li[2,*]

1.State Key Lab of New Ceramic and Fine Processing, School of Materials Science & Engineering, Tsinghua University, Beijing 100084, CHINA

2.Key Lab of Advanced Materials (MOE), School of Materials Science and Engineering, Tsinghua University, Beijing 100084, CHINA

3.Department of Engineering Physics, Tsinghua University, Beijing 100084, CHINA

* Corresponding author, Tel.: +86 10 61572233; fax: +86 10 61571160; Email address: zcli@tsinghua.edu.cn (Z. C. Li)

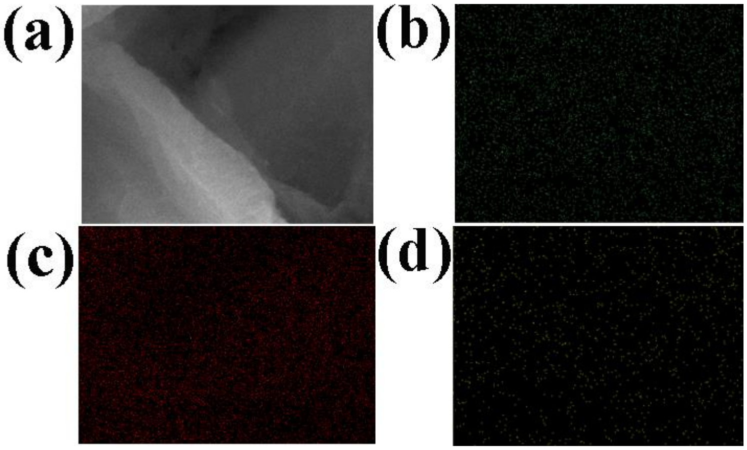

Figure S1: (a) EF-SEM images of 45s-Ag@ZnO-MNWAs; Element mappings of (b) Zn; (c) O; (d) Ag

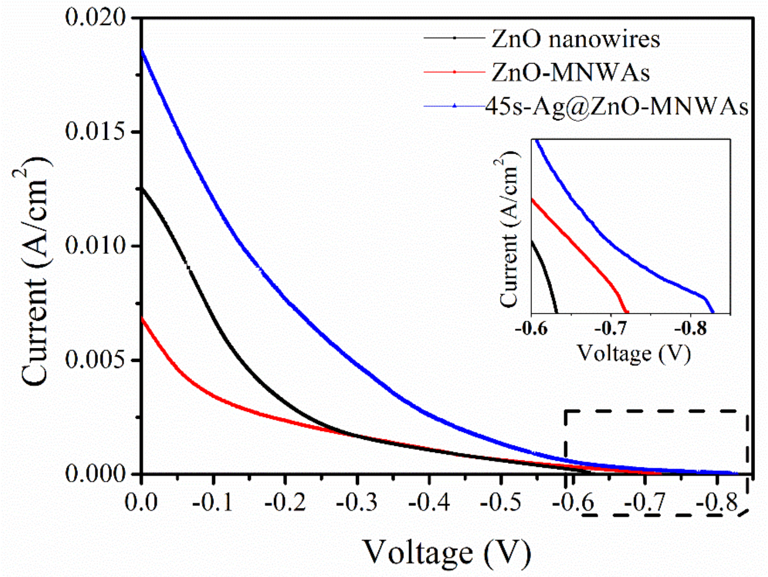

Figure S2: Photocurrent density-voltage curves of ZnO nanowires and ZnO-MNWAs and 45s-Ag@ZnO-MNWAs

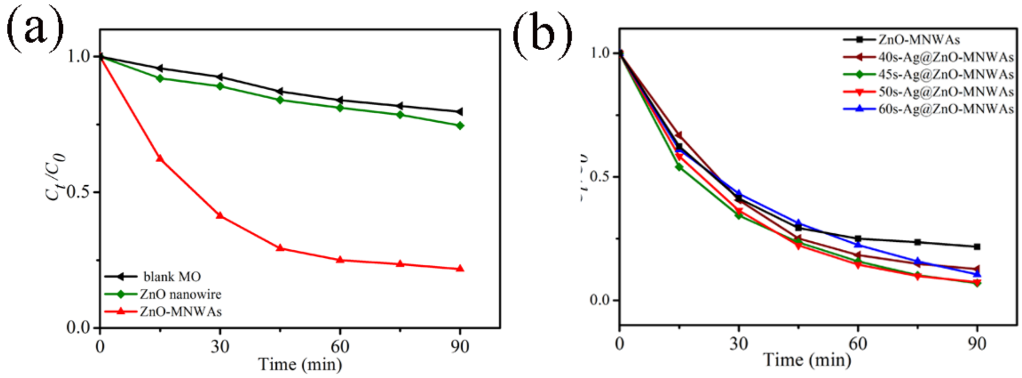

Figure S3: The photocatalytic activity of MO by (a) ZnO nanowires and ZnO-MNWAs, (b) ZnO-MNWAs modifying with different doses of Ag NPs.

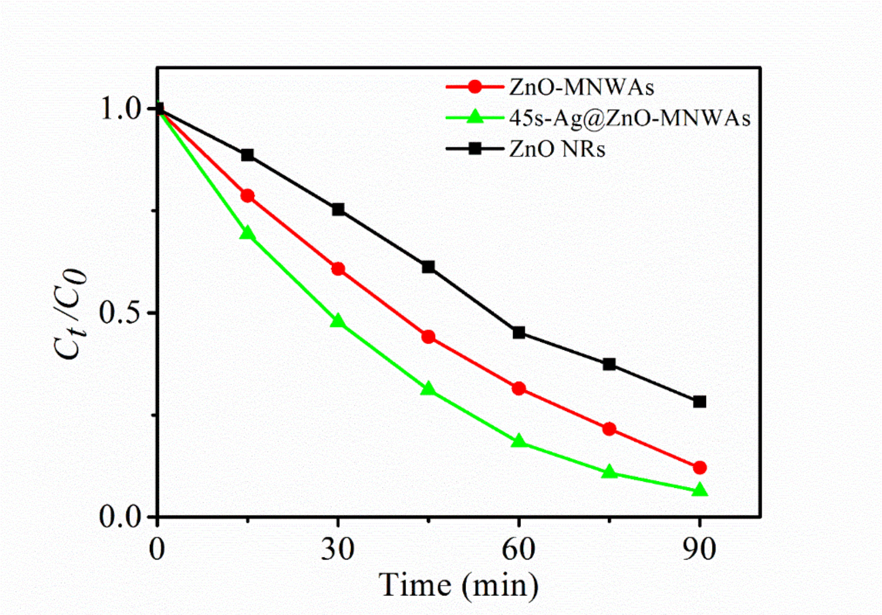

Figure S4: The photocatalytic activity of MC by ZnO nanowires and ZnO-MNWAs and 45s-Ag@ZnO-MNWAs

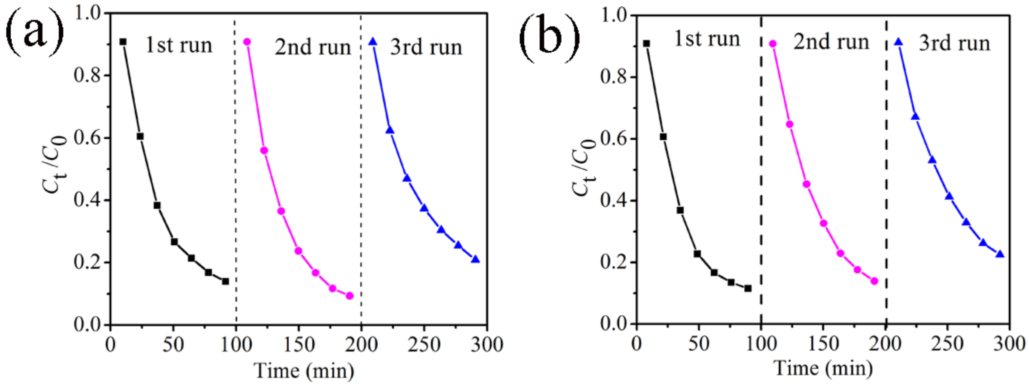

Figure S5: Cycling experiments of (a) ZnO-MNWAs, (b) 45s-Ag@ZnO-MNWAs
